# OPFR removal by white rot fungi: screening of removers and approach to the removal mechanism

**DOI:** 10.3389/ffunb.2024.1387541

**Published:** 2024-05-17

**Authors:** Diana Losantos, Montserrat Sarra, Glòria Caminal

**Affiliations:** ^1^ Department of Chemical, Biological and Environmental Engineering, Universitat Autònoma de Barcelona, Escola d’Enginyeria, Cerdanyola del Vallès, Spain; ^2^ Institut de Quiímica Avançada de Catalunya (IQAC), Spanish Council for Scientific Research (CSIC), Barcelona, Spain

**Keywords:** flame retardants, bioremediation, emerging pollutants, biosorption, fungi

## Abstract

The persistent presence of organophosphate flame retardants (OPFRs) in wastewater (WW) effluents raises significant environmental and health concerns, highlighting the limitations of conventional treatments for their remotion. Fungi, especially white rot fungi (WRF), offer a promising alternative for OPFR removal. This study sought to identify fungal candidates (from a selection of four WRF and two Ascomycota fungi) capable of effectively removing five frequently detected OPFRs in WW: tributyl phosphate (TnBP), tributoxy ethyl phosphate (TBEP), trichloroethyl phosphate (TCEP), trichloro propyl phosphate (TCPP) and triethyl phosphate (TEP). The objective was to develop a co-culture approach for WW treatment, while also addressing the utilization of less assimilable carbon sources present in WW. Research was conducted on carbon source uptake and OPFR removal by all fungal candidates, while the top degraders were analyzed for biomass sorption contribution. Additionally, the enzymatic systems involved in OPFR degradation were identified, along with toxicity of samples after fungal contact. Acetate (1.4 g·L^-1^), simulating less assimilable organic matter in the carbon source uptake study, was eliminated by all tested fungi in 4 days. However, during the initial screening where the removal of four OPFRs (excluding TCPP) was tested, WRF outperformed Ascomycota fungi. *Ganoderma lucidum* and *Trametes versicolor* removed over 90% of TnBP and TBEP within 4 days, with *Pleorotus ostreatus* and *Pycnoporus sanguineus* also displaying effective removal. TCEP removal was challenging, with only *G. lucidum* achieving partial removal (47%). A subsequent screening with selected WRF and the addition of TCPP revealed TCPP’s greater susceptibility to degradation compared to TCEP, with *T. versicolor* exhibiting the highest removal efficiency (77%). This observation, plus the poor degradation of TEP by all fungal candidates suggests that polarity of an OPFR inversely correlates with its susceptibility to fungal degradation. Sorption studies confirmed the ability of top-performing fungi of each selected OPFR to predominantly degrade them. Enzymatic system tests identified the CYP450 intracellular system responsible for OPFR degradation, so reactions of hydroxylation, dealkylation and dehalogenation are possibly involved in the degradation pathway. Finally, toxicity tests revealed transformation products obtained by fungal degradation to be more toxic than the parent compounds, emphasizing the need to identify them and their toxicity contributions. Overall, this study provides valuable insights into OPFR degradation by WRF, with implications for future WW treatment using mixed consortia, emphasizing the importance of reducing generated toxicity.

## Introduction

After the ban on polybrominated diphenyl ether flame retardants from the European Union (Lian et al., 2021; Xu et al., 2021), organophosphate flame retardants (OPFRs) have gained popularity due to their fire-inhibiting effectiveness. Halogenated OPFRs are now incorporated as additives in various commercial products, including furniture, electronics, foams, plastics, and textiles, while non-halogenated OPFRs are used as plasticizers and antifoaming agents (Shi et al., 2016; Zhang et al., 2021). Consequently, numerous OPFRs have become high-production-volume chemicals (USEPA, 2023), with a global production estimated at 2,800,000 tons in 2018 (Gustavsson et al., 2018) and are now considered emergent pollutants.

Since they lack chemical bonds with their host materials, OPFRs can readily diffuse into water compartments during production, usage, and end-of-life stages (Gbadamosi et al., 2023; Li et al., 2023), domestic and industrial wastewater (WW) discharges being the primary sources of contamination (Xu et al., 2019; Zhang et al., 2023). Among the prevalent compounds found in wastewater treatment plant (WWTP) effluents are five OPFRs: tri-n-butyl phosphate (TnBP), tris (2-butoxy ethyl) phosphate (TBEP), tris (2-chloroethyl) phosphate (TCEP), tris (2-chloroisopropyl) phosphate (TCPP), and triethyl phosphate (TEP) (Lao et al., 2023; Zhang et al., 2023), proving that they are not completely removed by conventional treatment processes.

Their extensive usage, persistence, and presence also in drinking water samples (Gbadamosi et al., 2023; Huang et al., 2023) have raised significant concerns in recent years. Thus, numerous studies have focused on their impact on diverse organisms. TnBP is known to be neurotoxic and irritating (Ahire et al., 2012; Kulkarni et al., 2014), while TBEP exhibits hepatotoxic and carcinogenic properties (Liu et al., 2022; Saquib et al., 2022). TEP has shown potential neurotoxic and mutagenic effects at high doses (Lai et al., 2022). Both TCEP and TCPP are considered carcinogenic and neurotoxic (Xu et al., 2017; Liu et al., 2022).

Fungal bioremediation, particularly by white rot fungi (WRF) is a promising, cost-effective, and eco-friendly approach for removing OPFRs. These microorganisms possess a sophisticated enzymatic system, which is non-specific and non-selective, and is therefore capable of constitutively breaking down a series of contaminants, even at trace concentrations. This system primarily comprises extracellular ligninolytic enzymes, such as laccase, manganese peroxidase lignin peroxidase, and versatile peroxidase (Manavalan et al., 2015), that degrade substrates by generating radicals (Asgher et al., 2008; Mir-Tutusaus et al., 2018a). The utilization of redox mediators produced for electron transfer can improve degradation, in the case of non-phenolic pollutants (Kunamneni et al., 2008). In many cases, their versatile cytochrome P450 intracellular system, consisting of a superfamily of monooxygenases, is responsible for the degradation of the contaminants (Marco-Urrea et al., 2006). Apart from enzymatic degradation processes, sorption onto the fungal biomass can also serve as a significant removal mechanism.

WRF generally degrade recalcitrant pollutants through a co-metabolic process. Since emergent pollutants are usually found at low concentrations in wastewater streams, they are not sufficient to support the metabolic requirements of the fungi by themselves (Beltrán-Flores et al., 2023). Therefore, the presence of a carbon source utilizable by WRF is essential in wastewater streams. However, these matrixes often contain various dissolved organic and inorganic compounds, that might not be accessible to WRF and in the worst case can affect their growth and inhibit their catalytic performance (Asif et al., 2017).

The main objective of this work was to identify fungal candidates capable of effectively removing water-soluble OPFRs, while using organic matter present in wastewater. The screening included four WRF, well-studied by the group (Palli et al., 2017; Jaén-Gil et al., 2019; Beltrán-Flores et al., 2021; Tan et al., 2023), but also two Ascomycota fungi. These Ascomycota fungi, selected from the *Trichoderma* and *Aspergillus* genera, also produce extracellular ligninolytic enzymes (laccase and peroxidases) (Dhakar et al., 2015; Kurkina et al., 2021), have demonstrated high efficiency in removing contaminants (Tripathi et al., 2013; Mukherjee, 2016), and possess a range of other enzymes such as oxidases, cellulases, xylanases, and amylases, which could potentially contribute to the degradation of either the OPFRs or other complex constituents in wastewater. The evaluation of these fungal candidates could allow the future use of a mixed consortium for wastewater treatment, promoting a co-culture approach that enhances enzymatic activities and promotes contaminant removal (Lira-Pérez et al., 2020).

The initial phase of the study concerned a carbon source uptake study. This step was crucial to determine whether the fungal candidates could utilize natural carbon sources present in wastewater. The final aim for this is to avoid the need for additional carbon sources during the treatment of a real effluent. Acetate was selected as the carbon source to simulate organic matter commonly found in wastewater (Yang et al., 2019), as it presents metabolic requirements different from those of glucose, making it less readily assimilable (Kutscha and Pflügl, 2020).

Subsequently, the removal efficiencies of these candidates in eliminating TEP, TBP, TBEP, and TCEP were evaluated. Those demonstrating efficient removal of acetate and these OPFRs were selected for further experiments. Then, the removal of the other chlorinated OPFRs, TCPP, and the effect of its addition on the removal efficiency of the other OPFRs were evaluated.

Additionally, the study aimed to investigate the mechanisms underlying OPFR removal. This involved evaluating the contribution of biomass sorption to removal and elucidating the enzymatic systems responsible for degradation. Finally, the toxicity of samples obtained after fungal contact was assessed to determine the efficacy of the treatment and ascertain whether the obtained transformation products were more toxic than the parent compounds, as observed in some cases (Cruz-Morató et al., 2013b).

## Materials and methods

### Reagents

High purity standards of OPFRs, namely, tri-n-butyl phosphate (TnBP ≥99%), tris(2-butoxy ethyl) phosphate (TBEP 94%), tris(2-chloroethyl) phosphate (TCEP 97%), triethyl phosphate (TEP ≥99.8%), and a mixture of isomers (TCPP) containing 66.9% of tris(1-chloro-2-propyl) phosphate (TCIPP/TCPP-IS1), 26.4% of bis(1-chloro-2-propyl) (2-chloropropyl) phosphate (TCPP-IS2), and 4.2% of (1-chloro-2-propyl) bis(2-chloropropyl) phosphate (TCPP-IS3) were purchased from Merck KGaA (Darmstadt, Germany). Methanol (MeOH) and dichloromethane (DCM) of chromatographic grade were obtained from Fisher Scientific (NH, USA) and Merck, respectively. A first stock solution containing OPFRs at concentrations of ~10,000 mg L^−1^ for TnBP and TBEP and ~5,000 mg L^−1^ for TEP and TCEP in methanol was prepared for the initial screening. A second stock solution, following the same procedure as the first, included TCPP at a concentration of ~5,000 mg L^−1^ and was used for all subsequent experiments. Both solutions were stored in the dark at −20°C until required.

D (+)-Glucose (C_6_H_12_O_6_) was purchased from Acros Organics (NJ, USA). Ammonium tartrate dibasic [(NH_4_)_2_C_4_H_4_O_6_] and 2,2-dimethyl succinic acid (C_6_H_10_O_4_) were obtained from Merck, as well as commercial laccase purified from *T. versicolor* (20 AU mg^-1^), laccase mediator 2,2′-Azino-bis (3-ethylbenzothiazoline-6-sulfonic acid) diammonium salt (ABTS), and cytochrome P450 inhibitor 1-Aminobenzotriazole (ABT). Anhydrous sodium acetate, used for acetate addition was acquired from Scharlab (Barcelona, Spain). Other high-purity grade chemicals were used for subculture media and as macronutrients and micronutrients of the defined medium.

### Microorganisms and media

The present study utilized four WRF: *Trametes versicolor, Ganoderma lucidum, Pycnoporus sanguineus*, and *Pleorotus ostreatus*, along with two Ascomycota fungi: *Aspergillus niger* and *Trichoderma viride* as fungal candidates. *Pleorotus ostreatus* CECT20311, *Aspergillus niger* CECT2807, and *Trichoderma viride* CECT20101 were sourced from the Spanish Type Culture Collection of the Universitat de València (Valencia, España). *Trametes versicolor* ATCC 42530 was obtained from the American Type Culture Collection (VA, USA), while *Ganoderma lucidum* FP-58537-Sp was acquired from the United States Department of Agriculture Collection (WI, USA). *Pycnoporus sanguineus* CS43 was generously supplied by the Environmental Bioprocesses Group of the Institute of Technology and Higher Studies of Monterrey (NL, México).

Strains aside from *A. niger* were maintained through subculturing on malt extract agar plates at 25°C every 30 days (Blánquez et al., 2008). Pellets were prepared in malt extract, following methodology described elsewhere (Romero et al., 2006). Briefly, 1 mL of homogenized mycelial suspension was inoculated in 1,000-mL Erlenmeyer flasks containing 250 mL of 2% malt extract medium (pH 4.5). Flasks were incubated for 7 days at 25°C under continuous orbital agitation (135 rpm). Afterward, fungal biomass was separated from the medium by means of a strainer and then diluted in a 0.80% (w/v) NaCl solution at a ratio of 1:1 (V_biomass_/V_NaCl_). The resultant suspension was stored at 4°C under sterile conditions, until use. In the case of *A. niger*, the same steps were performed, using potato dextrose agar for subculturing (Universitat de Valencia, 2024) and 2.4% of potato dextrose medium for pellets formation.

A defined medium was used for fungal maintenance during screening experiments, containing (per liter): 8 g of glucose, 3.3 g of ammonium tartrate, 1.168 g of dimethyl succinate, 10 mL of a micronutrient solution, and 100 mL of a macronutrient solution (Gros et al., 2014) from Kirk medium (Kirk et al., 1978). The composition of the nutrient solutions is specified in **Supplementary Table S1**. The pH of the medium was initially adjusted to 4.5 by means of NaOH and HCl solutions.

### Carbon source uptake

To test whether the studied fungi were able to use sodium acetate as the carbon source, its removal was compared with the removal of commonly used glucose, when TCEP is also present in the medium. Accordingly, the defined medium previously mentioned was modified. In this case, glucose and ammonium tartrate concentrations were reduced to 4 g·L^−1^ and 0.49 g·L^−1^, respectively, while 1.4 g·L^−1^ of sodium acetate was added. The acetate concentration was chosen so that the medium would have an initial pH of 4.5 without the need for additional pH regulation solutions, given the buffering properties of this salt. Glucose concentration was reduced to 4 g·L^−1^, to ensure that the combined amounts of glucose and acetate would equate to the minimum concentration of carbon source required for fungal maintenance (0.31 ± 0.03 g DCW^−1^ d^−1^; DCW=dry cell weight) (Blánquez et al., 2007). The ammonium tartrate concentration was adjusted to a carbon/nitrogen (C/N) ratio of 30 mol/mol, a minimally tested ratio at which there was optimal enzyme production for a week (time of the experiment) (Mir-Tutusaus et al., 2018b). These conditions were chosen to evaluate the fungi’s carbon uptake under the minimal requirements for their maintenance. TCEP was also added at a concentration of 10 mg·L^−1^.

Fungal pellets from each microorganism were inoculated at a concentration of ~2.5 g DCW·L^−1^ into 500-mL Erlenmeyer flasks containing 100 mL of the modified sterile defined medium. The cultures were incubated at 25°C with continuous orbital shaking (135 rpm) for 7 days. Each set of trials was conducted in triplicate. Samples (2 mL) were collected after 4 and 7 days (at the end of the experiment) to evaluate glucose and acetate concentrations, and soluble Chemical Oxygen Demand (COD).

### Fungal selection for OPFR removal

An initial screening of fungal removers was conducted using a sterile defined medium. The concentration of the pellets of each tested fungus was ~3.5 g DCW·L^−1^. The medium was enriched with the first stock solution until achieving concentrations of ~10 mg·L^−1^ for TnBP and TBEP and ~5 mg·L^−1^ for TEP and TCEP. Although these concentrations far exceed those reported in previous studies on WWTP effluents (the highest amount detected was 0.01 mg·L^−1^) (Pantelaki and Voutsa, 2019), they were selected for facilitating OPFR quantification. TEP and TCEP concentrations were lower than TnBP and TBEP as preliminary experiments (data not displayed) indicated that they might be more difficult to remove. The Erlenmeyer flasks were kept at 25°C, under continuous shaking, and in the absence of light to eliminate the impact of photodegradation on the removal. Abiotic flasks were used as controls. To assess the influence of the glucose regime, samples were collected at the beginning and after 4 days of the experiment to evaluate immediate removal under optimal glucose conditions. At day 4, glucose was re-supplemented at a concentration of 3 g/L. After 15 days (end of the experiment), the fungi were under nutrient-limiting conditions, and samples were collected again. Each set of trials was run in triplicate.

Finally, the proficiency of targeted candidates in removing a fifth OPFR, TCPP (known for its chlorinated nature), was evaluated. TCPP was added at a concentration of ~5 mg·L^−1^, along with the previously tested contaminants, to assess its impact on the removal of the other components. The experimental conditions and sampling times remained consistent with those of the screening. For both cases, 2-mL samples were withdrawn at the designated sampling times for the analysis of residual OPFR concentrations.

### Sorption contribution in OPFR removal

Samples were collected at the end of OPFR removal tests of each targeted candidate, to evaluate the extent of the degradation versus the sorption of the OPFRs on the fungal biomass. Thus, after taking the aliquot for GC-MS determination, the entire contents of one of the flasks of each triplicate were subjected to filtration, to separate the biomass from the liquid sample. This was done for each of the tested fungi. Vacuum filtration, utilizing fiberglass filters (1.6 µm, Whatman GF/A) was employed for this purpose. Both solid and liquid samples were preserved at −20°C until measurement.

### Evaluation of the enzymatic system involved in OPFR degradation

To determine the role of the extracellular and intracellular enzymatic systems in the degradation of the contaminants, both *in vitro* laccase-mediated and *in vivo* experiments with fungal pellets were performed by using *T. versicolor* as the model strain. The evaluation of the implication of each enzymatic system was determined according to the variation of the OPFR concentration throughout the experiments.

Laccase-mediated experiments were conducted using 250-mL Erlenmeyer flasks with 50 mL of a sodium malonate dibasic monohydrate solution (250 mM, pH 4.5) containing laccase from *T. versicolor* (Mir-Tutusaus et al., 2014). Laccase enzymatic activity was ~1,000 UA/L, as verified by the oxidation of 2,6-dimethoxiphenol (see laccase activity section). This solution was spiked with the stock solution, reaching concentrations of 10 mg·L^−1^ for TnBP and TBEP, and 5 mg·L^−1^ for TEP, TCEP, and TCPP. The effect of the addition of a laccase mediator, ABTS (0.8 mM), on the degradation of the contaminants was also evaluated (Marco-Urrea et al., 2009). On the other hand, the degradative capabilities of a biomass-free broth with laccase (pH 4.5; activity of 1,000 AU/L) were tested. Previously, *T. versicolor* pellets had been inoculated in this medium for 7 days to determine whether metabolites generated by the fungus itself could act as mediators for laccase. Abiotic controls (no laccase) were also conducted. All tested conditions were run simultaneously in triplicate. The flasks were incubated for 27 h at 25°C on an orbital shaker (135 rpm). At specified times, 0.9-mL aliquots were collected and mixed with 100 μL of 1-M HCl to stop the enzymatic reaction.

Regarding the involvement of the cytochrome P450 enzymes (CYP450) in OPFR degradation, a sterile-defined medium was fortified with the contaminants at concentrations equivalent to those used in laccase-mediated experiments. *T. versicolor* pellets were then inoculated at a concentration of ~3.5 g DCW·L^−1^. Under the same experimental conditions, the effect of the addition of ABT (5mM), an inhibitor of the CYP450 system was also examined (Marco-Urrea et al., 2009). Abiotic controls (without fungi and inhibitors) were conducted. All tested conditions were run simultaneously in triplicate. The flasks were kept in the dark for 15 days at 25°C on an orbital shaker (135 rpm). At specific intervals, 1-mL aliquots were collected.

### Toxicity determination

Toxicity assessment was conducted for samples collected at the beginning and end of the second fungal screening. Prior to testing, the pH of the samples was adjusted to 7. The test was carried out using an acute toxicity bioassay kit (Modern Water, London, UK), which relies on the attenuation of *Vibrio fischeri* bacteria bioluminescence following a 15-min exposure to selected dilutions of each sample. Toxicity levels were expressed in terms of toxicity units (TU), which are the reciprocal of the toxicant concentration at which 50% of the bacteria are inhibited at the end of the test (EC50), as displayed in Equation 1.


(1) $$TU=\;\frac{100}{EC50}\;$$

Inhibition at the highest toxicant concentration (81.9% of the initial concentration; allowed by the applied method) was also calculated according to Equations 2 and 3 (Abdallah et al., 2017).


(2) $$Inhibition\;\left(\%\right)=100-\left(\frac{I_{T}}{CF\;*\;I_{0}}\right)$$

(3) $$CF=\frac{IC_{n}}{IC_{0}}\;$$

Where

IT_n,_ luminescence of the test sample after the exposure time;

IT_0,_ initial luminescence of the test sample;

CF, correction factor, equivalent to;

IC_n_, luminescence of the control sample after the exposure time; and

IC_0,_ initial luminescence of the control sample.

### Analytical methods

#### Sample preparation and storage

Following collection, all samples, except the ones taken for the determination of the sorption contribution (refer to the section specific to this part), underwent filtration using syringe-driven filters to eliminate biomass. Polytetrafluoroethylene (PTFE) filters (0.2 µm, Millipore Millex-LG, Merck KGaA) were employed for glucose, soluble COD, and OPFR quantification during both the screening and evaluation of the enzymatic system. Meanwhile, Polyvinylidene difluoride (PVDF) filters (0.22 µm, Branchia) were used prior to acetate quantification. The filtered samples were frozen until measurement, except for glucose, which was determined right after sampling.

#### Glucose quantification

Glucose was measured by means of a biochemistry analyzer (2900 select; Yellow Springs Instrument, OH, USA).

#### Acetate quantification

Acetate analysis was conducted on a high-performance liquid chromatograph (HPLC Ultimate 3000; Dionex, Thermo Fisher Scientific, CA, USA), equipped with a refractive index detector (1260 Infinity; Agilent Technologies, CA, USA) and an autosampler. The chromatographic separation was carried out on an ICSep ICE-COREGEL 87H3 column (300 mm height, 7.8 mm diameter; Transgenomic, NE, USA). The column’s temperature was maintained at 25°C. The mobile phase was sulfuric acid 6 mM at a constant flow rate of 0.5 mL·min^−1^. Total run time was 30 min, and the retention time of acetate was approximately 18.31 min. The injection volume was 20 µL.

#### Soluble COD quantification

Soluble COD was determined by means of commercial kit LCK 014 (Hach Lange, IA, USA). Briefly, 0.5 mL of the sample was added to an LCK 014 cuvette containing a solution of sulfuric acid and potassium dichromate, as well as silver and mercury ions. Silver works as a catalyst, while mercury is used to complex chloride interferences. Following mixing, the sample was heated for 2 h at 148°C. After cooling at room temperature, the amount of Cr^+3^ formed through the reduction of the dichromate ion, because of the presence of organic compounds, was measured using a colorimetric method, with a DR3900 VIS Benchtop Spectrophotometer (Hach Lange, IA, USA). The test wavelength was 620 nm.

#### Laccase activity

Laccase activity was determined through the oxidation of 2,6-dimethoxiphenol (DMP) (Cruz-Morató et al., 2013a). The reaction mixture comprised 0.6 mL of 250-mM sodium malonate at pH 4.5, 150 µL of 20-mM DMP, and 1.8 mL of the sample, resulting in a total volume of 2.55 mL. Absorbance changes at 468 nm were recorded for 2 min on a Varian Cary 3 UV/Vis spectrophotometer at a temperature of 30°C. Laccase activity was quantified in AU/L, where an activity unit (AU) was defined as micromoles of DMP oxidized per minute. The molar extinction coefficient for DMP was 24,800 M^−1^ cm^−1^ (Wariishi et al., 1992).

#### OPFR quantification during the screening and evaluation of the enzymatic system

Concentrations of the tested OPFRs were determined by mass spectrometry, following a liquid–liquid extraction with DCM. After extraction, the organic phase (containing the contaminant) was separated from the medium. Such phase was directly injected from the vial to a GC-MS. Equipment and analytical conditions are detailed in the work of Losantos et al. (2024).

#### OPFR quantification for sorption experiments

##### Extraction in the solid phase

Biomass samples were subjected to freeze-drying and then extraction with 15 mL of a hexane:acetone (1:1 v/v) mixture. An ultrasound-assisted extraction (UAE) was then performed for 15 min, followed by centrifugation at 3,220 RCF for 5 min. UAE and centrifugation were repeated twice, and the resulting extracts were collected and evaporated to dryness at 20°C under a nitrogen stream. Subsequently, 5 mL of a hexane:methanol (1:3) mixture was introduced, followed by an additional centrifugation step under the same conditions as mentioned earlier. A 200-µL aliquot was then collected and spiked with 15 µL of a mixture comprising Internal Standards (IS; initial concentration=1 µg/mL each) for all the tested OPFRs.

##### Preparation of the aqueous fraction

In a similar fashion to the solid samples, the aqueous fraction was composed of the liquid sample and a small amount of internal standard (IS). Thus, in a 25-mL volumetric flask, 12 mL of the liquid sample was added, followed by the addition of 50 µL of each IS (initial concentration = 50 µg/mL). Sample addition continued until the flask was filled to the capacity mark. An aliquot was taken and filtered through syringe filters (0.2 µm, PTFE, Interchim, Montluçon, France) prior to chromatographic analysis.

##### Chromatographic analysis

Analytical performance was assessed using a TurboFlow™ TFC-LC-MS/MS provided by Thermo Fisher Scientific (Waltham, MA, USA). Sample purification was carried out in two combined LC columns, Cyclone™-P (0.5 mm × 50 mm) and C18-XL (0.5 mm × 50 mm), while chromatographic separation was performed in a Purosphere Star RP-18 (125 mm × 0.2 mm, particle size 5 µm) analytical column. Mobile phases were performed in gradient elution for both purification (TFC) and analytical (LC) steps, with water (0.1% formic acid) and methanol (0.1% formic acid) solutions for TFC at a flow rate of 0.75 mL min^−1^, and water (0.1% formic acid) and methanol (ammonium acetate, 10 mM) solutions for LC at 0.25 mL min^−1^. Instrumental conditions were previously described (Giulivo et al., 2016).

## Results

### Carbon source uptake

In this section, the ability of the tested fungi to use sodium acetate in the presence of 10 ppm of TCEP was evaluated. Sodium acetate served as a less assimilable carbon source to the fungi, in comparison to the commonly used glucose. Consequently, both carbon sources were quantified following simultaneous addition into a defined medium containing each fungus in pelletized form, after 4 and 7 days.

The removal percentages for each carbon source are displayed in **Supplementary Table S2**. All fungi demonstrated the ability to assimilate both carbon sources within 4 days. *T. versicolor* was excluded from assessment in this instance, as Beltrán-Flores et al. (2023) reported that the ATCC 42530 strain could completely remove twice the amount of acetate in approximately 5.5 days compared to the concentration used in this study. Although this test was performed without the addition of any pollutant, we anticipate this ability to remain unchanged, given the results obtained in our study. The research also highlighted a notably faster consumption of glucose compared to acetate, as an equivalent amount of glucose was utilized after 24 h, attributed to a more energetically efficient assimilation. It is likely that this holds true for all the tested fungi, as in our case, glucose was supplemented at ~3 times more than acetate.

Nonetheless, when looking at soluble COD removals (refer to **Supplementary Figure S1**), it is evident that complete removal is impossible. Theoretical COD calculations indicated that the primary contributor to COD was methanol coming from the initial stock used for TCEP addition, where TCEP was at a concentration of 1,000 mg·L^−1^. Methanol (theoretical COD = 11,860 mg·L^−1^) accounted for approximately 60% of the initial soluble COD. This proportion closely aligns with the non-removed fraction observed in the results, suggesting that the tested fungi are incapable of removing the methanol present in the medium. In consequence, subsequent tests involving OPFRs were performed using stock solutions of 10,000 mg·L^−1^ and 5,000 mg·L^−1^, as outlined in the Materials and methods section.

### Fungal selection for OPFR removal

**Figure 1:**
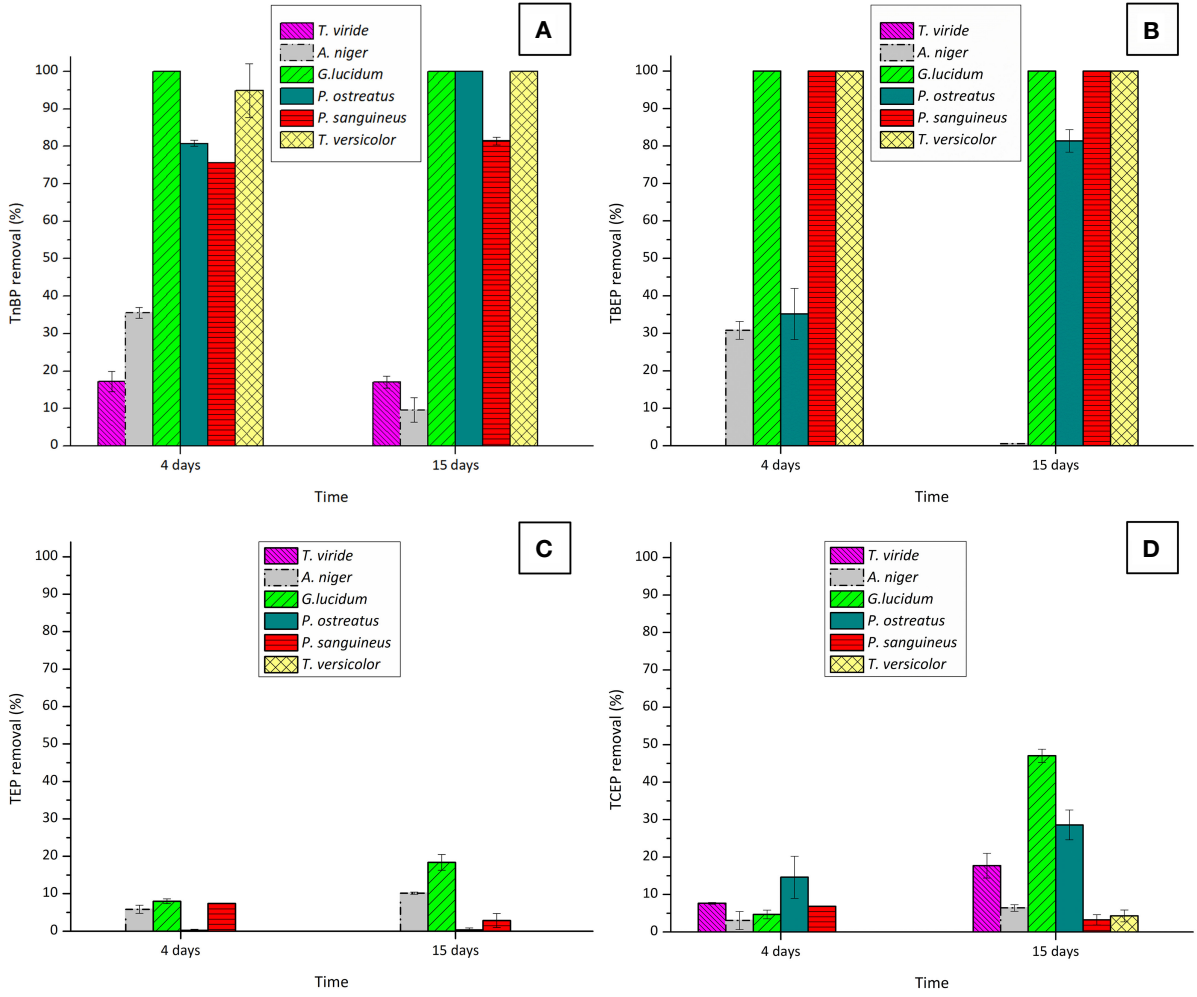
Removal percentages of the OPFRs: **(A)** TnBP, **(B)** TBEP, **(C)** TEP, and **(D)** TCEP after 4 and 15 days of fungal contact with six different fungi. Initial concentrations were of 10 mg·L^−1^ for TnBP and TBEP and 5 mg·L^−1^ for TEP and TCEP.

An initial screening was conducted to select fungi demonstrating optimal performance in removing OPFRs. Removal percentages achieved for each OPFR following each fungal treatment are depicted in **Figure 1**.

These initial findings highlight the superior efficacy of WRF in removing these pollutants, in comparison with the Ascomycota fungi *T. viride* and *A. niger*. This difference in performance is likely attributed to significant variations in the enzymatic systems of each fungus, although belonging to the same superfamily. These variations might result in different roles in the remotion of contaminants (Chen et al., 2014). In the case of *T. viride*, a decrease in the biomass dry weight was observed at the end of the experiment (data not shown), possibly due to the defined medium used not providing the necessary nutrients for the fungus viability (Iqbal et al., 2017). Additionally, examining the removal behavior of TnBP and TBEP after 4 and 15 days of fungal contact with *A. niger*, reveals a decline between these two time points. This phenomenon is more likely associated with a desorption process from *A. niger’s* fungal biomass. In fact, this fungus has been widely used for the removal of heavy metals (Faez and Jebur Al-Mamoori, 2021; Chau et al., 2023) due to its sorption capacities. Considering the overall performance of the tested Ascomycota fungi for OPFR removal, performing further studies only with WRF was decided.

In evaluating the efficacy of the WRF for removing TnBP and TBEP, both *G. lucidum* and *T. versicolor* demonstrated remarkable efficiency, by removing more than 90% of these compounds within a very short period (4 days). This proficiency is consistent with findings from other studies where this ability by the fungi to remove complex xenobiotics was tested (Beltrán-Flores et al., 2022; Shokrollahzadeh et al., 2023; Tan et al., 2023). Although exhibiting lower overall performances, *P. ostreatus* and *P. sanguineus* are also strong candidates for the removal of these OPFRs.

The removal of TCEP was only partial, thus highlighting the complexity of this chlorinated compound. While *P. ostreatus* achieved a partial removal of TCEP (29%), *G. lucidum* emerged as the most effective candidate, with removal increasing to 47%. The removal was greatly enhanced when the fungus experienced a shortage of the carbon source. This nutrient-deprivation approach has already been identified as inducing the WRF degradative system in some cases (Reddy and Mathew, 2001; Gupta et al., 2017).

Interestingly, concerning TEP (the non-chlorinated form of TCEP), the removal efficiency of *G. lucidum* was unexpectedly low, reaching only 20%. This observation led to the hypothesis that the polarity of the tested OPFRs has a significant role in their remotion by fungal species, as TEP has a higher polarity than TCEP (refer to **Supplementary Table S3**).

**Figure 2:**
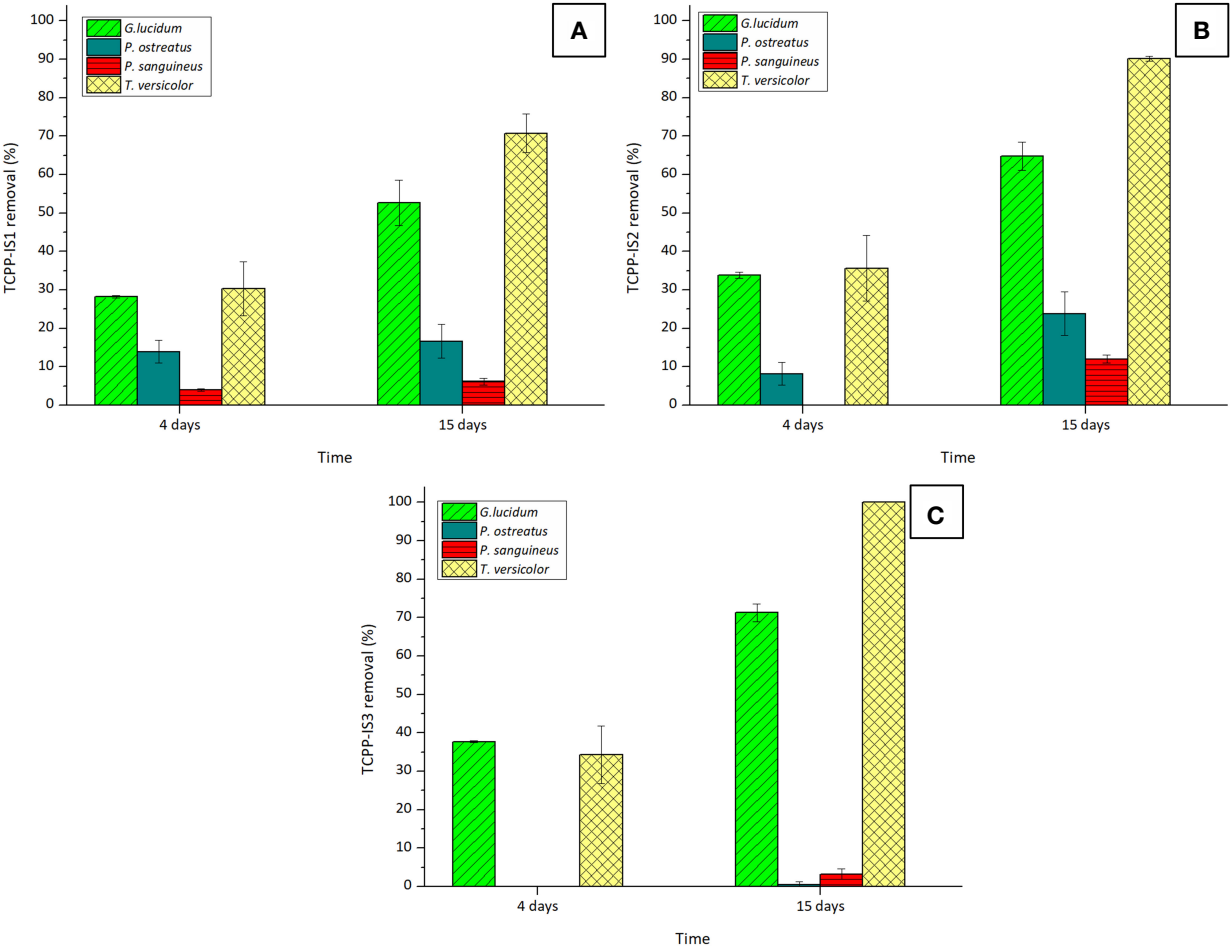
Removal percentages of isomers: **(A)** TCPP-IS1, **(B)** TCPP-IS2, and **(C)** TCPP-IS3 of TCPP by the four tested WRF after 4 and 15 days of experiment. Initial concentration of TCPP was 5 mg·L^−1^.

To corroborate this hypothesis, the remotion of the chlorinated compound TCPP (mixture of three isomers), which is less polar than TCEP, was also examined. The outcomes are depicted in **Figure 2**.

As expected, TCPP exhibited greater susceptibility to removal than TCEP, with *T. versicolor* emerging as the most efficient fungus in breaking down this pollutant. However, complete remotion of this compound was not possible, attributed once again to the presence of chlorine molecules in the isomers’ structures. Overall removal of this compound with *T. versicolor*, considering the contribution of the three isomers was 77.01% ± 3.66%.

The impact of the presence of TCPP on the elimination of the other examined OPFRs was also investigated. **Table 1** illustrates the variations in removal percentages for all the OPFRs across the tested fungal matrixes.

**Table 1:** Variations in removal percentages of previously tested OPFRs, 15 days after the addition of TCPP.

Compound	*G. lucidum*	*P. ostreatus*	*P. sanguineus*	*T. versicolor*
TnBP	0.00 ± 0.00^a^	-3.26 ± 0.90^c^	**−12.25 ± 0.11^c^**	0.00 ± 0.00^a^
TBEP	0.00 ± 0.00^a^	2.26 ± 0.74^b^	0.00 ± 0.00^a^	0.00 ± 0.00^a^
TEP	−10.49 ± 2.14^c^	−0.36 ± 0.51^c^	−2.84 ± 1.86^c^	0.00 ± 0.00^a^
TCEP	−11.11 ± 5.04^c^	**−27.93 ± 3.04^c^**	−3.01 ± 1.03^c^	−3.47 ± 0.46^c^

Numbers marked with ^a^ signify no variation; numbers with ^b^ indicate an increase, and numbers with ^c^ signify a decrease in removal. Numbers in bold highlight significant variations (p-value <0.05 at a 95% confidence interval).

The decrease in TCEP removal was evident, with removal decreasing to 36% in the case of *G. lucidum*, while *P. ostreatus* completely lost its ability to remove this OPFR. This pattern is also observed in the case of TEP, providing further support for the earlier hypothesis and suggesting a general principle: the more polar an OPFR, the less susceptible it is to removal by WRF. This behavior aligns with observations in other biological treatments (Liang and Liu, 2016). We theorize that when degrading the contaminants, WRF take radicals from them (Barr and Aust, 1994), making longer chains more available to the fungus and consequently more easily broken.

It is also worth mentioning that *P. sanguineus* not only is the least effective remover of TCPP, but also experiences an overall efficiency decrease in the presence of this contaminant. This might be related to inhibitory effects. Nonetheless, no information regarding the toxicity of TCPP on fungal species has been found, so further research should be performed to clarify this behavior.

Fungal removal of OPFRs is scarcely reported, while research has been focusing mainly on bacterial removal. For instance, TBEP at a concentration of 5 mg·L^−1^ was completely removed by a culture enriched from activated sludge, where *Rhodocuccus ruber* strain C1 was identified as the key degrader (Liang et al., 2023). Although mineralization was not achieved, significant elimination of TBEP was also attained by a sludge culture, with removal percentages of 83.3%, for low-starting concentrations (0.3 mg·L^−1^) and 66.7% for higher-starting concentrations (3 mg·L^−1^). TnBP was also partially removed by this culture, although at lower extents of 75% for low-starting concentrations and 41.6% for high-starting concentrations. The importance of the enrichment step for microbial remotion of these contaminants was highlighted, as culture without enrichment displayed a comparably decreased performance (Nancharaiah et al., 2015; Hou et al., 2021). On the other hand, a complete mineralization of TnBP by an enriched consortium from aerobic granular biomass has been reported. The high removal rates obtained for TBEP and TBnP in our study, comparable to those reported in literature, further underscore the proficiency of WRF in removing these contaminants.

As of 2017, *Sphingobium* sp. strain TCM1 and *Sphingomonas* sp. strain TDK1 were the only reported TCEP-degrading microorganisms (Takahashi et al., 2017). Both strains were able to mineralize ~5.71 mg·L^−1^ of TCEP over different time periods (Takahashi et al., 2010). In recent years, Rhizobiales strains have emerged as significant candidates for the complete mineralization of 50 mg·L^−1^ TCEP by enriched sediment microbial communities (Zhou et al., 2020; Liang et al., 2022). Consequently, the knowledge about TCEP potential degraders remains limited, making the evaluation of *G. lucidum* as a fungal candidate for TCEP removal (although mineralization was not achieved) an important step toward expanding the spectrum of potential TCEP degraders.

Treatment of TCPP posed challenges when treated with a strain of *Providencia rettgeri* isolated from a polluted sediment, as only 34.7% of 1 mg·L^−1^ TCPP were decomposed (Ye et al., 2024). Conversely, complete remotion of TCPP at the same concentration was achieved by strain *Amycolatopsis* sp. FT-1, after 6 months of culture enrichment from a sludge–water mixture (Feng et al., 2023). However, when the concentration of TCPP was increased to 5 mg · L^−1^, remotion decreased to 85.3%, similar to that achieved by *T. versicolor* at this concentration, suggesting its potential as a candidate for TCPP removal.

Finally, it is noteworthy that the reported literature on bacterial remediation of OPFRs entails an initial enrichment step to enable efficient degradation by bacteria. In contrast, the advantage of WRF lies in their ability to degrade these contaminants constitutively. Additionally, most studies in the literature focus on bacterial efficiency in removing a single OPFR, whereas our study represents the first in-depth report on fungal remediation of a mixture of OPFRs, which more accurately reflects their presence in wastewater.

### Sorption contribution in OPFR removal

**Figure 3:**
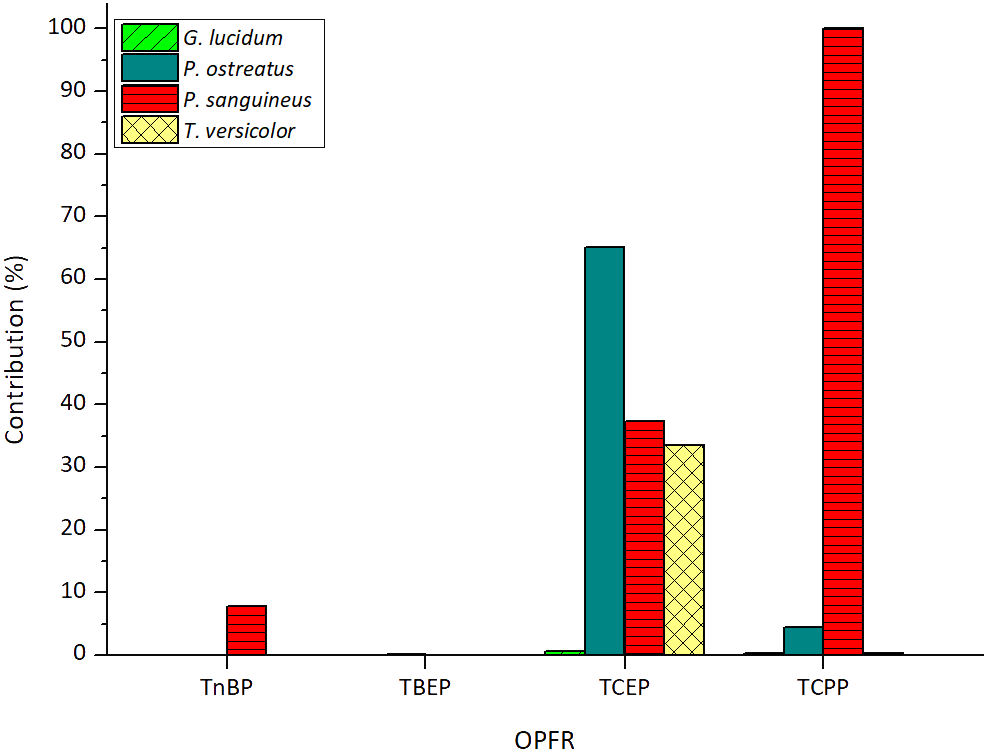
Sorption contributions of each tested fungus for the removal of OPFRs. The sorbed amount of TCPP encompasses the contribution of its three main isomers.

Biosorption onto fungal biomass can be a significant mechanism for removing xenobiotics, and its contribution should be studied to determine its extent against degradation. Sorption contributions of each fungus for the removal of the tested OPFRs are depicted in **Figure 3**. The sorption of TEP was not observed in *G. lucidum* matrix and thus, was not included in the figure. This suggests that the small amount removed was completely degraded.

As observed in the figure, both TnBP and TBEP exhibit a very low sorption effect, which means high susceptibility to degradation by all the tested fungi. Regarding TBEP, sorption contributions were consistently below 0.23% across all cases, resulting in an average contribution of 0.07% ± 0.10%. For TnBP, sorption contributions were below 0.06% in three out of the four tested fungi, with *P. sanguineus* also displaying a low contribution of 7.81%. Consequently, an average contribution of less than 5% was determined.

For the chlorinated compounds, average contributions are not reported due to significant variations in the sorption contributions of each fungus. In the case of TCEP, *G. lucidum* is the only fungus that nearly entirely degrades the removed percentage of this contaminant with a low sorption contribution of 0.67%. The fact that this fungus is the only efficient degrader of TCEP in the presence of TCPP further reinforces its suitability for treating this contaminant. A similar behavior is consistent for *T. versicolor* and *G. lucidum* in the case of TCPP, with sorption contributions of 0.28% and 0.37%, respectively.

It is noteworthy that *P. sanguineus* is unable to metabolize TCPP as the small amount that is removed from the medium is completely biosorbed. This emphasizes the notion of inhibition of the fungus by the presence of this contaminant. On the other hand, considering this strain’s notable feature of possessing a highly active and stable laccase enzymatic activity (Ramírez-Cavazos et al., 2014), it is probable that this enzyme does not serve as the main catalyst for TCPP degradation, an aspect that will be explored in the next section.

### Evaluation of the enzymatic system involved in OPFR degradation

Both *in vitro* laccase-mediated and *in vivo* experiments with fungal pellets were performed using *T. versicolor* as the model strain. This choice was based on the fungus’ efficient degradation of two out of three of the non-chlorinated compounds, and its status as the best candidate for TCPP degradation (refer to **Figure 2**). Additionally, *Trametes* is a remarkable laccase producer (Wang et al., 2016).

#### Influence of laccase

The contribution of different laccase-mediated conditions on OPFR degradation is outlined in **Supplementary Table S4**.

The results show that laccase alone was unable to break down any of the tested OPFRs, as expected, due to the impossibility of a direct interaction between these compounds and the enzyme, owing to their non-phenolic nature (Palli et al., 2016). The presence of the mediator ABTS, although expected to help the reaction take place, also proved ineffective.

Interestingly, in the presence of the mediator, laccase managed to degrade 20% of the TCEP present in the medium. It has been previously indicated that the presence of chloride can induce competitive inhibition in *T. versicolor* laccase (Raseda et al., 2014), suggesting that chloride uptake from TCEP might hinder the degradation of the other OPFRs. However, results obtained with laccase in the broth obtained after fungal contact indicate that metabolites secreted by the WRF fail to promote oxidation of these contaminants.

#### Influence of the CYP450 system

**Figure 4:**
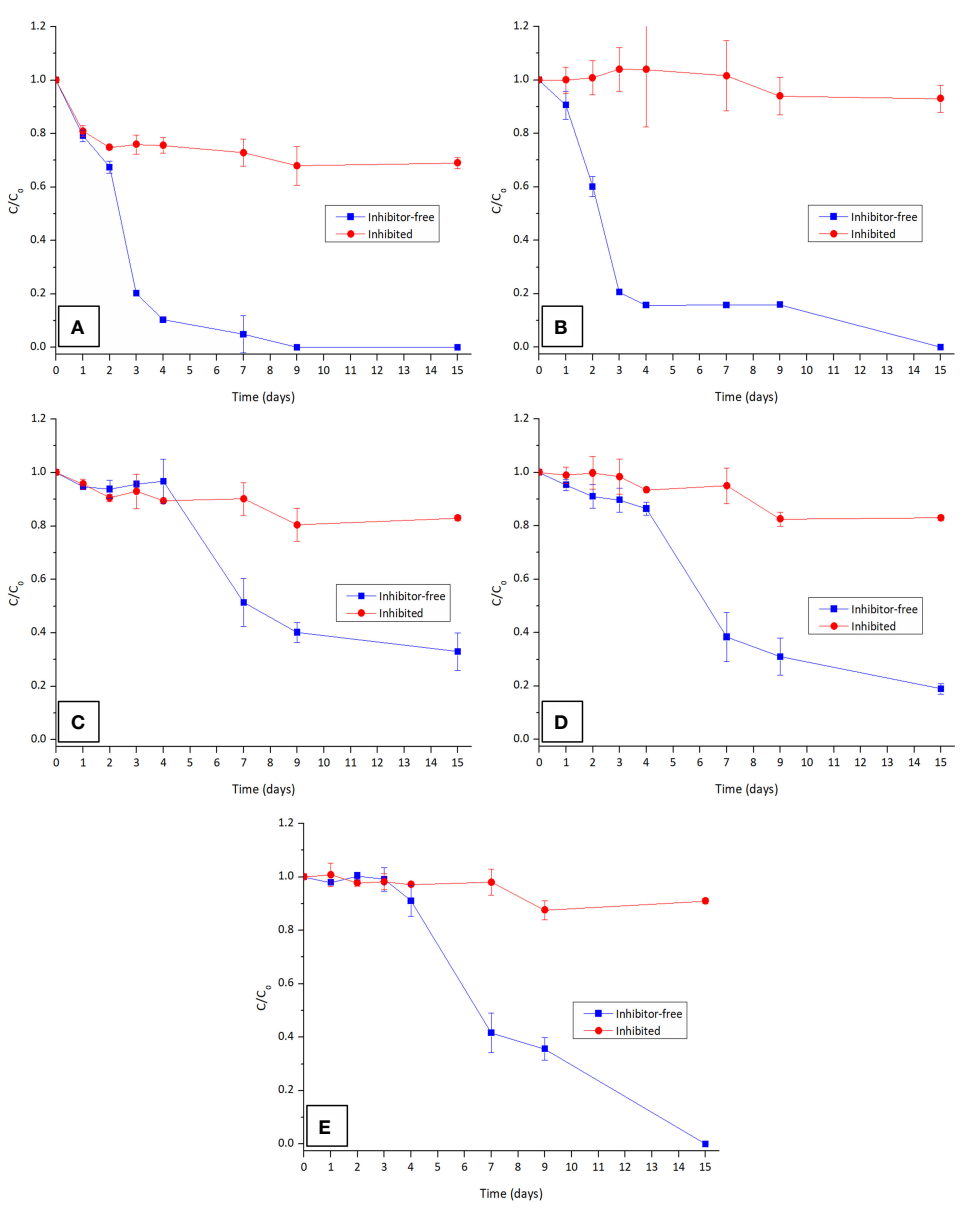
Contribution of CYP450 in the degradation of the OPFRs: **(A)** TnBP, **(B)** TBEP, isomers **(C)** TCPP-IS1, **(D)** TCPP-IS2, and **(E)** TCPP-IS3 in the presence and absence of CYP450 inhibitor ABT. Both TEP and TCEP are not presented as they are not degraded by T. versicolor.

The involvement of the CYP450 system in the degradation of the tested OPFRs was assessed by comparing the degradation efficiency of *T. versicolor* under inhibitor-free conditions, against the addition of ABT, a well-known inhibitor of this system. The results are presented in **Figure 4**.

As anticipated, the presence of the inhibitor considerably slowed down the degradation rate for all the OPFRs, underscoring the significant role of the CYP450 enzymes in the degradation of these compounds by WRF. Although the contribution of the CYP450 system for the degradation of TCEP could not be determined, it is highly probable that the *G. lucidum* family of monooxygenases also plays a key role in degrading this contaminant. Metabolic reactions of hydroxylation, epoxidation, dealkylation, and dehalogenation are typically involved in the degradation of xenobiotics by this system and are likely implicated in the degradation pathway of the tested OPFRs.

This test results also facilitated the determination of a time-course degradation profile for these contaminants. It is evident that TnBP degrades more rapidly than TBEP, which again proves the relevance of polarity for the removal of these contaminants. Upon closer examination of TCPP isomers, although it seems that isomer 3 disappears faster than the other two, it is important to consider that this isomer is initially present at a very low concentration. In fact, isomer 1 undergoes a more rapid elimination compared to isomer 2, which, in turn, disappears faster than isomer 3 (refer to **Supplementary Figure S2**). Thus, degradation at these concentration levels adheres to pseudo-first-order kinetics.

### Toxicity determination

Toxicity was evaluated in terms of *Vibrio fischeri* bioluminescence inhibition. The obtained toxicity units at the start and end of each fungal removal experiment are displayed in **Table 2**. Inhibition at the highest toxicants’ concentrations allowed by the method can be found in **Supplementary Figure S3**.

**Table 2:** Toxicity of samples collected at the start and end of each fungal remotion experiment.

	Initial	*P. sanguineus*	*T. versicolor*	*G. lucidum*	*P. ostreatus*
Toxicity units (TU)	n.p.^*^	15.13 ± 0.11	7.35 ± 0.06	3.59 ± 0.11	n.p.^*^

*not provided as EC50 was superior to the tested concentrations, which means that no toxicity was detected at this level.

The initial sample, containing the five OPFRs, exhibited non-toxicity for *V. fischeri* at the tested concentrations. However, fungal treatment led to an increase in toxicity in all cases, most likely related to the transformation products obtained. Diesters produced from the five tested contaminants have been linked to various toxic effects (Kojima et al., 2016; Zhang et al., 2020; Liu et al., 2021), suggesting that these may also be generated during fungal degradation.

Transformation products obtained by three out of four WRF candidates inhibited more than 50% of the present bacteria, enabling the calculation of toxicity units. In the case of *P. ostreatus*, although the EC50 could not be determined, there was an increase in the maximum toxicity from 18%, obtained from the initial sample at time 0, to 29% (refer to **Supplementary Figure S3**).

Curiously, *P. sanguineus* generates the highest toxicity among the group, but is only able to degrade TnBP and TBEP, while *G. lucidum*, capable of degrading TCEP and TCPP, produces less toxicity. These differences suggest significant variations in the extent of the degradation pathway, emphasizing the importance of determining the transformation products from each OPFR, and their respective contributions to toxicity.

## Conclusions

The four WRF exhibited the capacity to remove OPFRs. Two key observations emerged: First, the more polar an OPFRs, the less susceptible it is to removal by WRF. Second, chlorinated compounds’ removal is significantly enhanced by nutrient deprivation. Low sorption contributions suggest that OPFRs are predominantly degraded by WRF, except for TCPP, which was entirely sorbed by *P. sanguineus* biomass. This, coupled with decreased removal efficiencies for the other OPFRs by this fungus, is indicative of inhibitory effects resulting from the presence of this contaminant. The involvement of the CYP450 intracellular enzymatic system in OPFR degradation suggests potential reactions of hydroxylation, epoxidation, dealkylation, and dehalogenation in the degradation pathway. However, the resultant transformation products prove to be more toxic than the parent compounds. These results not only represent a significant advancement in understanding the mechanisms and the risks associated with OPFR fungal removal, but they are also an incentive for testing alternative approaches to mitigate toxicity along the degradation pathway. In addition, all fungi displayed the capacity to assimilate both carbon sources (glucose and acetate) within 4 days in the presence of an OPFR. However, methanol from the stock solution remained unremoved, preventing the complete elimination of COD.

## Data availability statement

The original contributions presented in the study are included in the article/**Supplementary Material**. Further inquiries can be directed to the corresponding author.

## Author contributions

DL: Data curation, Formal analysis, Investigation, Methodology, Writing – original draft. MS: Conceptualization, Funding acquisition, Supervision, Writing – review & editing. GC: Conceptualization, Formal analysis, Methodology, Supervision, Writing – review & editing.

## References

[B1] Abdallah A. Daher A. Belghmi K. Yigerta Da D. Blaghen M. (2017). Detoxification assessment of inorganic mercury by bioluminescence of vibrio fischeri. Res. J. Environ. Toxicol. 11, 104–111. doi: 10.3923/rjet.2017.104.111

[B2] Ahire K. C. Kapadnis B. P. Kulkarni G. J. Shouche Y. S. Deopurkar R. L. (2012). Biodegradation of tributyl phosphate by novel bacteria isolated from enrichment cultures. Biodegradation 23, 165–176. doi: 10.1007/s10532-011-9496-7 21755325

[B3] Asgher M. Bhatti H. N. Ashraf M. Legge R. L. (2008). Recent developments in biodegradation of industrial pollutants by white rot fungi and their enzyme system. Biodegradation 19, 771–783. doi: 10.1007/s10532-008-9185-3 18373237

[B4] Asif M. B. Hai F. I. Hou J. Price W. E. Nghiem L. D. (2017). Impact of wastewater derived dissolved interfering compounds on growth, enzymatic activity and trace organic contaminant removal of white rot fungi – A critical review. J. Environ. Manage 201, 89–109. doi: 10.1016/j.jenvman.2017.06.014 28651223

[B5] Barr D. P. Aust S. D. (1994). Mechanisms white rot fungi use to degrade pollutants. Environ. Sci. Technol. 28, 78A–87A. doi: 10.1021/es00051a724 22662714

[B6] Beltrán-Flores E. Pla-Ferriol M. Martínez-Alonso M. Gaju N. Blánquez P. Sarrà M. (2022). Fungal bioremediation of agricultural wastewater in a long-term treatment: biomass stabilization by immobilization strategy. J. Hazard Mater 439, 129614. doi: 10.1016/j.jhazmat.2022.129614 35882168

[B7] Beltrán-Flores E. Sarrà M. Blánquez P. (2021). Pesticide bioremediation by Trametes versicolor: Application in a fixed-bed reactor, sorption contribution and bioregeneration. Sci. Total Environ. 794, 148386. doi: 10.1016/j.scitotenv.2021.148386 34218143

[B8] Beltrán-Flores E. Tayar S. Blánquez P. Sarrà M. (2023). Effect of dissolved oxygen on the degradation activity and consumption capacity of white-rot fungi. J. Water Process Eng. 55, 104105. doi: 10.1016/j.jwpe.2023.104105

[B9] Blánquez P. Caminal G. Sarrà M. Vicent T. (2007). The effect of HRT on the decolourisation of the Grey Lanaset G textile dye by Trametes versicolor. Chem. Eng. J. 126, 163–169. doi: 10.1016/j.cej.2006.09.007

[B10] Blánquez P. Sarrà M. Vicent T. (2008). Development of a continuous process to adapt the textile wastewater treatment by fungi to industrial conditions. Process Biochem. 43, 1–7. doi: 10.1016/j.procbio.2007.10.002

[B11] Chau T. P. Bulgariu L. Saravanan M. Rajkumar R. Chinnathambi A. Salmen S. H.. (2023). Bioremediation efficiency of free and immobilized form of Aspergillus Niger and Aspergillus tubigenesis biomass on tannery effluent. Environ. Res. 231, 116275. doi: 10.1016/j.envres.2023.116275 37257743

[B12] Chen W. Lee M. K. Jefcoate C. Kim S. C. Chen F. Yu J. H. (2014). Fungal cytochrome P450 monooxygenases: Their distribution, structure, functions, family expansion, and evolutionary origin. Genome Biol. Evol. 6, 1620–1634. doi: 10.1093/gbe/evu132 24966179 PMC4122930

[B13] Cruz-Morató C. Ferrando-Climent L. Rodriguez-Mozaz S. Barceló D. Marco-Urrea E. Vicent T.. (2013a). Degradation of pharmaceuticals in non-sterile urban wastewater by Trametes versicolor in a fluidized bed bioreactor. Water Res. 47, 5200–5210. doi: 10.1016/j.watres.2013.06.007 23866144

[B14] Cruz-Morató C. Jelić A. Perez S. Petrović M. Barceló D. Marco-Urrea E.. (2013b). Continuous treatment of clofibric acid by Trametes versicolor in a fluidized bed bioreactor: Identification of transformation products and toxicity assessment. Biochem. Eng. J. 75, 79–85. doi: 10.1016/j.bej.2013.03.020

[B15] Dhakar K. Kooliyottil R. Joshi A. Pandey A. (2015). Simultaneous production of ligninolytic enzymes by a temperature and pH tolerant strain of Aspergillus Niger under different cultural conditions. Indian J. Biotechnol. 14, 81–86.

[B16] Faez H. Jebur Al-Mamoori A. M. (2021). Cells immobilization of some microorganisms as a tool for bioremediation: b- Aspergillus Niger. IOP Conf Ser Earth Environ Sci. 722, 012009. doi: 10.1088/1755-1315/722/1/012009

[B17] Feng M. Xie Y. Mao W. Lu Y. Wang Y. Li H.. (2023). Efficient biodegradation of tris-(2-chloroisopropyl) phosphate by a novel strain Amycolatopsis sp. FT-1: Process optimization, mechanism studies and toxicity changes. J. Hazard Mater 443, 130149. doi: 10.1016/j.jhazmat.2022.130149 36252405

[B18] Gbadamosi M. R. Al-Omran L. S. Abdallah M. A. E. Harrad S. (2023). Concentrations of organophosphate esters in drinking water from the United Kingdom: Implications for human exposure. Emerg. Contam 9, 100203. doi: 10.1016/j.emcon.2023.100203

[B19] Giulivo M. Capri E. Eljarrat E. Barceló D. (2016). Analysis of organophosphorus flame retardants in environmental and biotic matrices using on-line turbulent flow chromatography-liquid chromatography-tandem mass spectrometry. J. Chromatogr A 1474, 71–78. doi: 10.1016/j.chroma.2016.10.042 27817833

[B20] Gros M. Cruz-Morato C. Marco-Urrea E. Longrée P. Singer H. Sarrà M.. (2014). Biodegradation of the X-ray contrast agent iopromide and the fluoroquinolone antibiotic ofloxacin by the white rot fungus Trametes versicolor in hospital wastewaters and identification of degradation products. Water Res. 60, 228–241. doi: 10.1016/j.watres.2014.04.042 24867600

[B21] Gupta S. Wali A. Gupta M. Annepu S. K. (2017). “Fungi: An effective tool for bioremediation,” in Plant-Microbe Interactions in Agro-Ecological Perspectives (Singapore: Springer), 593–606. doi: 10.1007/978-981-10-6593-4_24

[B22] Gustavsson J. Wiberg K. Ribeli E. Nguyen M. A. Josefsson S. Ahrens L. (2018). Screening of organic flame retardants in Swedish river water. Sci. Total Environ. 625, 1046–1055. doi: 10.1016/j.scitotenv.2017.12.281 29996401

[B23] Hou R. Wang Y. Zhou S. Zhou L. Yuan Y. Xu Y. (2021). Aerobic degradation of nonhalogenated organophosphate flame esters (OPEs) by enriched cultures from sludge: Kinetics, pathways, bacterial community evolution, and toxicity evaluation. Sci. Total Environ. 760, 143385. doi: 10.1016/j.scitotenv.2020.143385 33243516

[B24] Huang Q. Mao X. Pan F. Hu X. He Z. Wang Y.. (2023). Organophosphate esters in source, finished, and tap water in Wuhan, China. Chemosphere 325, 138288. doi: 10.1016/j.chemosphere.2023.138288 36871801

[B25] Iqbal S. Ashfaq M. Malik A. H. Inam-ul-haq M. Khan K. S. Mathews P.. (2017). Isolation, preservation and revival of Trichoderma Viride in culture media. J. Entomol Zool Stud. 5, 1640–1646.

[B26] Jaén-Gil A. Castellet-Rovira F. Llorca M. Villagrasa M. Sarrà M. Rodríguez-Mozaz S.. (2019). Fungal treatment of metoprolol and its recalcitrant metabolite metoprolol acid in hospital wastewater: Biotransformation, sorption and ecotoxicological impact. Water Res. 152, 171–180. doi: 10.1016/j.watres.2018.12.054 30669039

[B27] Kirk T. K. Schultz E. Connors W. J. Lorenz L. F. Zeikus J. G. (1978). Influence of culture parameters on lignin metabolism by Phanerochaete chrysosporium. Arch. Microbiol. 117, 277–285. doi: 10.1007/BF00738547

[B28] Kojima H. Takeuchi S. Van den Eede N. Covaci A. (2016). Effects of primary metabolites of organophosphate flame retardants on transcriptional activity via human nuclear receptors. Toxicol. Lett. 245, 31–39. doi: 10.1016/j.toxlet.2016.01.004 26778350

[B29] Kulkarni S. Markad V. L. Melo J. S. D’Souza S. F. Kodam K. M. (2014). Biodegradation of tributyl phosphate using Klebsiella pneumoniae sp. S3. Appl. Microbiol. Biotechnol. 98, 919–929. doi: 10.1007/s00253-013-4938-2 23644771

[B30] Kunamneni A. Ghazi I. Camarero S. Ballesteros A. Plou F. J. Alcalde M. (2008). Decolorization of synthetic dyes by laccase immobilized on epoxy-activated carriers. Process Biochem. 43, 169–178. doi: 10.1016/j.procbio.2007.11.009

[B31] Kurkina Y. Travkin V. Solyanikova I. (2021). Biotechnological potential of fungi and bacteria with ligninolytic activity (mini-review). BIO Web Conf. 30, 05005. doi: 10.1051/bioconf/20213005005

[B32] Kutscha R. Pflügl S. (2020). Microbial upgrading of acetate into value-added products—examining microbial diversity, bioenergetic constraints and metabolic engineering approaches. Int. J. Mol. Sci. 21, 1–30. doi: 10.3390/ijms21228777 PMC7699770 33233586

[B33] Lai Y. J. Wang X. W. Liu J. F. (2022). Occurrence of trimethyl phosphate and triethyl phosphate in a municipal wastewater treatment plant and human urine. Environ. Pollutants Bioavailability 34, 146–153. doi: 10.1080/26395940.2022.2064338

[B34] Lao J. Y. Xu S. Zhang K. Lin H. Cao Y. Wu R.. (2023). New perspective to understand and prioritize the ecological impacts of organophosphate esters and transformation products in urban stormwater and sewage effluents. Environ. Sci. Technol. 57, 11656–11665. doi: 10.1021/acs.est.3c04159 37503546

[B35] Li W. Yuan Y. Wang S. Liu X. (2023). Occurrence, spatiotemporal variation, and ecological risks of organophosphate esters in the water and sediment of the middle and lower streams of the Yellow River and its important tributaries. J. Hazard Mater 443, 130153. doi: 10.1016/j.jhazmat.2022.130153 36244105

[B36] Lian M. Lin C. Wu T. Xin M. Gu X. Lu S.. (2021). Occurrence, spatiotemporal distribution, and ecological risks of organophosphate esters in the water of the Yellow River to the Laizhou Bay, Bohai Sea. Sci. Total Environ. 787, 147528. doi: 10.1016/j.scitotenv.2021.147528 33991915

[B37] Liang K. Liu J. (2016). Understanding the distribution, degradation and fate of organophosphate esters in an advanced municipal sewage treatment plant based on mass flow and mass balance analysis. Sci. Total Environ. 544, 262–270. doi: 10.1016/j.scitotenv.2015.11.112 26657372

[B38] Liang Y. Zhou X. Wu Y. Wu Y. Gao S. Zeng X.. (2022). Rhizobiales as the key member in the synergistic tris (2-chloroethyl) phosphate (TCEP) degradation by two bacterial consortia. Water Res. 218, 118464. doi: 10.1016/j.watres.2022.118464 35461102

[B39] Liang Y. Zhou X. Wu Y. Wu Y. Zeng X. Yu Z.. (2023). Meta-omics elucidates key degraders in a bacterial tris(2-butoxyethyl) phosphate (TBOEP)-degrading enrichment culture. Water Res. 233, 119774. doi: 10.1016/j.watres.2023.119774 36848852

[B40] Lira-Pérez J. Rodríguez-Vázquez R. Chan-Cupul W. (2020). Effect of fungal co-cultures on ligninolytic enzyme activities, H _2_ O _2_ production, and orange G discoloration. Prep Biochem. Biotechnol. 50, 607–618. doi: 10.1080/10826068.2020.1721534 32013716

[B42] Liu Y. Gong S. Ye L. Li J. Liu C. Chen D.. (2021). Organophosphate (OP) diesters and a review of sources, chemical properties, environmental occurrence, adverse effects, and future directions. Environ. Int. 155, 106691. doi: 10.1016/j.envint.2021.106691 34146766

[B41] Liu M. Li A. Meng L. Zhang G. Guan X. Zhu J.. (2022). Exposure to novel brominated flame retardants and organophosphate esters and associations with thyroid cancer risk: A case-control study in eastern China. Environ. Sci. Technol. 56, 17825–17835. doi: 10.1021/acs.est.2c04759 36468700

[B43] Losantos D. Palacios O. Berge M. J. Sarrà M. Caminal G. Eustaquio A. (2024). Novel method for rapid monitoring of OPFRs by LLE and GC–MS as a tool for assessing biodegradation: validation and applicability. Anal. Bioanal Chem. 416, 1493–1504. doi: 10.1007/s00216-024-05154-7 38280016 PMC10861394

[B44] Manavalan T. Manavalan A. Heese K. (2015). Characterization of lignocellulolytic enzymes from white-rot fungi. Curr. Microbiol. 70, 485–498. doi: 10.1007/s00284-014-0743-0 25487116

[B45] Marco-Urrea E. Gabarrell X. Sarrà M. Caminal G. Vicent T. Reddy C. A. (2006). Novel aerobic perchloroethylene degradation by the white-rot fungus Trametes versicolor. Environ. Sci. Technol. 40, 7796–7802. doi: 10.1021/es0622958 17256530

[B46] Marco-Urrea E. Pérez-Trujillo M. Vicent T. Caminal G. (2009). Ability of white-rot fungi to remove selected pharmaceuticals and identification of degradation products of ibuprofen by Trametes versicolor. Chemosphere 74, 765–772. doi: 10.1016/j.chemosphere.2008.10.040 19062071

[B47] Mir-Tutusaus J. A. Baccar R. Caminal G. Sarrà M. (2018a). Can white-rot fungi be a real wastewater treatment alternative for organic micropollutants removal? A review. Water Res. 138, 137–151. doi: 10.1016/j.watres.2018.02.056 29579480

[B48] Mir-Tutusaus J. A. Caminal G. Sarrà M. (2018b). Influence of process variables in a continuous treatment of non-sterile hospital wastewater by Trametes versicolor and novel method for inoculum production. J. Environ. Manage 212, 415–423. doi: 10.1016/j.jenvman.2018.02.018 29455149

[B49] Mir-Tutusaus J. A. Masís-Mora M. Corcellas C. Eljarrat E. Barceló D. Sarrà M.. (2014). Degradation of selected agrochemicals by the white rot fungus Trametes versicolor. Sci. Total Environ. 500–501, 235–242. doi: 10.1016/j.scitotenv.2014.08.116 25217998

[B50] Mukherjee A. (2016). “Role of Aspergillus in Bioremediation Process,” in New and Future Developments in Microbial Biotechnology and Bioengineering. (Amsterdam: Elsevier), 209–214. doi: 10.1016/B978-0-444-63505-1.00017-8

[B51] Nancharaiah Y. V. Kiran Kumar Reddy G. Krishna Mohan T. V. Venugopalan V. P. (2015). Biodegradation of tributyl phosphate, an organosphate triester, by aerobic granular biofilms. J. Hazard Mater 283, 705–711. doi: 10.1016/j.jhazmat.2014.09.065 25464313

[B52] Palli L. Castellet-Rovira F. Pérez-Trujillo M. Caniani D. Sarrà-Adroguer M. Gori R. (2017). Preliminary evaluation of Pleurotus ostreatus for the removal of selected pharmaceuticals from hospital wastewater. Biotechnol. Prog. 33, 1529–1537. doi: 10.1002/btpr.2520 28653347

[B53] Palli L. Gullotto A. Tilli S. Caniani D. Gori R. Scozzafava A. (2016). Biodegradation of 2-naphthalensulfonic acid polymers by white-rot fungi: Scale-up into non-sterile packed bed bioreactors. Chemosphere 164, 120–127. doi: 10.1016/j.chemosphere.2016.08.071 27587355

[B54] Pantelaki I. Voutsa D. (2019). Organophosphate flame retardants (OPFRs): A review on analytical methods and occurrence in wastewater and aquatic environment. Sci. Total Environ. 649, 247–263. doi: 10.1016/j.scitotenv.2018.08.286 30173033

[B1001] Ramírez-Cavazos L. I. Junghanns C. Ornelas-Soto N. Cárdenas-Chávez D. L. Hernández-Luna C. Demarche P.. (2014). Purification and characterization of two thermostable laccases from Pycnoporus sanguineus and potential role in degradation of endocrine disrupting chemicals. J Mol Catal B Enzym. 108, 32–42. doi: 10.1016/j.molcatb.2014.06.006

[B55] Raseda N. Hong S. Yul Kwon O. Ryu K. (2014). Kinetic evidence for the interactive inhibition of laccase from trametes versicolor by ph and chloride. J. Microbiol. Biotechnol. 24, 1673–1678. doi: 10.4014/jmb.1408.08012 25152059

[B56] Reddy C. A. Mathew Z. (2001). “Bioremediation potential of white rot fungi,” in Fungi in bioremediation (Cambridge: Cambridge University Press), 52–78. Available at: http://www.britmycolsoc.org.uk.

[B57] Romero S. Blánquez P. Caminal G. Font X. Sarrà M. Gabarrell X.. (2006). Different approaches to improving the textile dye degradation capacity of Trametes versicolor. Biochem. Eng. J. 31, 42–47. doi: 10.1016/j.bej.2006.05.018

[B58] Saquib Q. Al-Salem A. M. Siddiqui M. A. Ansari S. M. Zhang X. Al-Khedhairy A. A. (2022). Tris(2-butoxyethyl) phosphate (TBEP): A flame retardant in solid waste display hepatotoxic and carcinogenic risks for humans. Chemosphere 296, 133977. doi: 10.1016/j.chemosphere.2022.133977 35216979

[B59] Shi Y. Gao L. Li W. Wang Y. Liu J. Cai Y. (2016). Occurrence, distribution and seasonal variation of organophosphate flame retardants and plasticizers in urban surface water in Beijing, China. Environ. pollut. 209, 1–10. doi: 10.1016/j.envpol.2015.11.008 26618261

[B60] Shokrollahzadeh S. Tayar S. Azizmohseni F. Safavi M. Keypour S. (2023). Fungal decolorization of toxic Triphenylmethane dye by newly isolated Ganoderma fungi: Growth, enzyme activity, kinetics. Bioresour Technol. Rep. 24, 101654. doi: 10.1016/j.biteb.2023.101654

[B61] Takahashi S. Katanuma H. Abe K. Kera Y. (2017). Identification of alkaline phosphatase genes for utilizing a flame retardant, tris(2-chloroethyl) phosphate, in Sphingobium sp. strain TCM1. Appl. Microbiol. Biotechnol. 101, 2153–2162. doi: 10.1007/s00253-016-7991-9 27866252

[B62] Takahashi S. Satake I. Konuma I. Kawashima K. Kawasaki M. Mori S.. (2010). Isolation and identification of persistent chlorinated organophosphorus flame retardant-degrading bacteria. Appl. Environ. Microbiol. 76, 5292–5296. doi: 10.1128/AEM.00506-10 20525857 PMC2916485

[B63] Tan Z. Losantos D. Li Y. Sarrà M. (2023). Biotransformation of chloramphenicol by white-rot-fungi Trametes versicolor under cadmium stress. Bioresour Technol. 369, 128508. doi: 10.1016/j.biortech.2022.128508 36549514

[B64] Tripathi P. Singh P. C. Mishra A. Chauhan P. S. Dwivedi S. Bais R. T.. (2013). Trichoderma: A potential bioremediator for environmental clean up. Clean Technol. Environ. Policy 15, 541–550. doi: 10.1007/s10098-012-0553-7

[B65] Universitat de Valencia. (2024). CECT 2807 strain data. Available at: https://www.cect.org/vstrn.php?lan=es&cect=2807.

[B66] USEPA. (2023). High Production Volume List. Available at: https://comptox.epa.gov/dashboard/chemical-lists/EPAHPV (Accessed November 2, 2023).

[B67] Wang K.-F. Hu J.-H. Guo C. Liu C.-Z. (2016). Scale-up laccase production from Trametes versicolor stimulated by vanillic acid. Bioprocess Biosyst. Eng. 39, 1041–1049. doi: 10.1007/s00449-016-1582-0 26971792

[B68] Wariishi H. Valli K. Gold M. H. (1992). Manganese(II) oxidation by manganese peroxidase from the basidiomycete Phanerochaete chrysosporium. Kinetic mechanism and role of chelators. J. Biol. Chem. 267, 23688–23695. doi: 10.1016/S0021-9258(18)35893-9 1429709

[B69] Xu L. Hu Q. Liu J. Liu S. Liu C. Deng Q.. (2019). Occurrence of organophosphate esters and their diesters degradation products in industrial wastewater treatment plants in China: Implication for the usage and potential degradation during production processing. Environ. pollut. 250, 559–566. doi: 10.1016/j.envpol.2019.04.058 31026704

[B71] Xu T. Li P. Wu S. Lei L. He D. (2017). Tris(2-chloroethyl) phosphate (TCEP) and tris(2-chloropropyl) phosphate (TCPP) induce locomotor deficits and dopaminergic degeneration in Caenorhabditis elegans. Toxicol. Res. (Camb) 6, 63–72. doi: 10.1039/C6TX00306K 30090477 PMC6060632

[B70] Xu L. Zhang B. Hu Q. Liu Y. Shang T. Zeng X.. (2021). Occurrence and spatio-seasonal distribution of organophosphate tri- and di-esters in surface water from Dongting Lake and their potential biological risk. Environ. pollut. 282, 117031. doi: 10.1016/j.envpol.2021.117031 33831629

[B72] Yang J. Li J. Zheng Z. Hou L. Liang D. Sun Y.. (2019). Effect of organic matters on anammox coupled denitrification system: When nitrite was sufficient. R Soc. Open Sci. 6, 190771. doi: 10.1098/rsos.190771 31827829 PMC6894598

[B73] Ye J. Tang S. Qiu R. Chen S. Liu H. (2024). Biodegradation pathway and mechanism of tri (2-chloropropyl) phosphate by Providencia rettgeri. J. Environ. Sci. (China) 144, 26–34. doi: 10.1016/j.jes.2023.07.023 38802235

[B76] Zhang Y. Cui W. Zhang N. Qin P. Zhang Y. Guo X.. (2023). Occurrence and risk of organophosphate flame retardants in multiple urban water of beijing, China. Water Air Soil pollut. 234, 1–10. doi: 10.1007/s11270-023-06277-w

[B75] Zhang S. Li Y. Yang C. Meng X. Z. Zheng H. Gao Y.. (2021). Application of Hi-throat/Hi-volume SPE technique in analyzing occurrence, influencing factors and human health risk of organophosphate esters (OPEs) in drinking water of China. J. Environ. Manage 291, 112714. doi: 10.1016/j.jenvman.2021.112714 33940361

[B74] Zhang Q. Yu C. Fu L. Gu S. Wang C. (2020). New insights in the endocrine disrupting effects of three primary metabolites of organophosphate flame retardants. Environ. Sci. Technol. 54, 4465–4474. doi: 10.1021/acs.est.9b07874 32150676

[B77] Zhou X. Liang Y. Ren G. Zheng K. Wu Y. Zeng X.. (2020). Biotransformation of tris(2-chloroethyl) phosphate (TCEP) in sediment microcosms and the adaptation of microbial communities to TCEP. Environ. Sci. Technol. 54, 5489–5497. doi: 10.1021/acs.est.9b07042 32264671

