# Supplementary material for: OPFR removal by white rot fungi: screening of removers and approach to the removal mechanism
Source: Front Fungal Biol. 2024 May 17;5:1387541. doi: 10.3389/ffunb.2024.1387541 (PMC11140845; doi:10.3389/ffunb.2024.1387541)
Supplement: Supplementary file 1 [file DataSheet_1.docx]

**Supporting information**

OPFRs removal by White Rot Fungi: Screening of removers and approach to the removal mechanism

Diana Losantos^a^, Montserrat Sarra^a,*^, Glòria Caminal^b^.

^a^ *Department of Chemical, Biological and Environmental Engineering, Universitat Autònoma de Barcelona, Escola d’Enginyeria, Campus Bellaterra 08193 Cerdanyola del Vallès, Spain*

^b^ *Institut de Química Avançada de Catalunya (IQAC), Spanish Council for Scientific Research (CSIC). Jordi Girona 18-26, 08034 Barcelona, Spain.*

^*^ Corresponding author email: [montserrat.sarra@uab.cat](mailto:montserrat.sarra@uab.cat)

D. Losantos ORCID: 0000-0002-7782-3537

M. Sarra ORCID: 0000-0002-3447-6328

G. Caminal ORCID: 0000-0001-9646-6099

**Table S. 1.** Composition of the micronutrients and macronutrients stock solution

| **Micronutrients** | | **Macronutrients** | |
| --- | --- | --- | --- |
| **Component** | **Concentration (g·L^-1^)** | **Component** | **Concentration (g·L^-1^)** |
| N(CH₂CO₂H)₃ | 1.5 | KH₂PO_4_ | 20.0 |
| MgSO_4_ · 7H_2_O | 3.0 | MgSO_4_ · 7H_2_O | 5.0 |
| MnSO_4_ · H_2_O | 0.5 | CaCl_2_ | 1.0 |
| NaCl | 1.0 |  |  |
| FeSO_4_ · 7H_2_O | 0.1 |  |  |
| CoSO_4_ | 0.1 |  |  |
| ZnSO_4_ · 7H_2_O | 0.1 |  |  |
| CaCl_2_ · 2H_2_O | 0.1 |  |  |
| CuSO_4_ · 5H_2_O | 0.01 |  |  |
| AlK(SO_4_)_2_ · 12H_2_O | 0.01 |  |  |
| H_3_BO_3_ | 0.01 |  |  |
| Na_2_MoO_4_ | 0.01 |  |  |

**Table S. 2:** Removal of each carbon source by the tested fungi after 4 and 7 days of experimentation*

|  | **Source of carbon removal (%)** | | | |  |
| --- | --- | --- | --- | --- | --- |
| **Strain** | **Glucose** | | **Acetate** | |  |
|  | **4 days** | **7 days** | **4 days** | **7 days** |  |
| *T. viride* | 99.68 ± 0.28 | 99.78 ± 0.19 | 100.00 ± 0.00 | 100.00 ± 0.00 |  |
| *A. niger* | | 99.97 ± 0.05 | 99.72 ± 0.25 | 100.00 ± 0.00 | 100.00 ± 0.00 |
| *G. lucidum* | 99.70 ± 0.08 | 100.00 ± 0.00 | 95.94 ± 0.34 | 97.77 ± 0.30 |  |
| *P. ostreatus* | 99.96 ± 0.05 | 99.83 ± 0.16 | 100.00 ± 0.00 | 100.00 ± 0.00 |  |
| *P. sanguineus* | 99.81 ± 0.26 | 99.90 ± 0.09 | 100.00 ± 0.00 | 100.00 ± 0.00 |  |

**Trametes versicolor* was not included in the carbon source uptake study as its efficiency to completely remove acetate has already been proven (Beltrán-Flores et al., 2023)





**Figure S. 1:** Soluble COD removals by the tested fungi after 4 and 7 days of experimentation.

**Table S. 3:** Physicochemical properties of OPFRs tested in this work.

| **Compound** | **Abbreviation** | **Water solubility (mg·L^-1^) at 25°C** | **log K_ow_** |
| --- | --- | --- | --- |
| Tributyl phosphate | TBP | 2.80 x 10^2^ | 4.00 |
| Tris(2-butoxy ethyl) phosphate | TBEP | 1.20 x 10^3^ | 3.65 |
| Tris(2-chloroethyl) phosphate | TCEP | 7.00 x 10^3^ | 1.44 |
| Tris(2-chloroisopropyl) phosphate | TCPP | 1.60 x 10^3^ | 2.59 |
| Triethyl phosphate | TEP | 5.00 x 10^5^ | 0.80 |

(*ChemSpider*, 2011; Lee et al., 2016; Van der Veen & de Boer, 2012)

**Table S. 4:** Contribution of different laccase-mediated conditions in OPFRs removal. Results are expressed as the ratio between concentrations obtained at specific time intervals and the initial concentration (C/C_0_). Laccase was obtained from T. versicolor. Enzimatic activity=1000 AU/L; ABTS (Mediator) initial concentration=0.8 mM; Medium was obtained after fungal contact for 7 days.

|  | **TBP** | | | **TBEP** | | |
| --- | --- | --- | --- | --- | --- | --- |
| **Time (h)** | **Laccase** | **Laccase + ABTS** | **Laccase + medium** | **Laccase** | **Laccase + ABTS** | **Laccase + medium** |
| **0** | 1.00 ± 0.00 | 1.00 ± 0.00 | 1.00 ± 0.00 | 1.00 ± 0.00 | 1.00 ± 0.00 | 1.00 ± 0.00 |
| **1** | 1.00 ± 0.01 | 1.01 ± 0.04 | 1.02 ± 0.01 | 0.97 ± 0.01 | 0.98 ± 0.01 | 1.05 ± 0.02 |
| **2.5** | 0.99 ± 0.03 | 0.99 ± 0.05 | 1.02 ± 0.04 | 0.95 ± 0.01 | 1.01 ± 0.01 | 1.04 ± 0.04 |
| **5** | 0.99 ± 0.04 | 1.02 ± 0.08 | 1.02 ± 0.00 | 1.01 ± 0.06 | 0.97 ± 0.02 | 1.00 ± 0.00 |
| **7.5** | 0.96 ± 0.02 | 0.97 ± 0.01 | 0.97 ± 0.00 | 1.00 ± 0.04 | 0.96 ± 0.01 | 1.00 ± 0.06 |
| **9** | 0.97 ± 0.00 | 0.98 ± 0.01 | 1.00 ± 0.04 | 0.99 ± 0.01 | 0.99 ± 0.05 | 1.03 ± 0.03 |
| **21** | 0.97 ± 0.01 | 1.03 ± 0.03 | 0.96 ± 0.01 | 0.99 ± 0.01 | 1.01 ± 0.06 | 1.05 ± 0.02 |
| **27** | 0.95 ± 0.03 | 0.94 ± 0.01 | 1.00 ± 0.01 | 1.00 ± 0.08 | 1.06 ± 0.01 | 0.99 ± 0.01 |
|  | **TEP** | | | **TCEP** | | |
| **Time (h)** | **Laccase** | **Laccase + ABTS** | **Laccase + medium** | **Laccase** | **Laccase + ABTS** | **Laccase + medium** |
| **0** | 1.00 ± 0.00 | 1.00 ± 0.00 | 1.00 ± 0.00 | 1.00 ± 0.00 | 1.00 ± 0.00 | 1.00 ± 0.00 |
| **1** | 1.00 ± 0.01 | 0.97 ± 0.02 | 1.03 ± 0.03 | 1.00 ± 0.00 | **0.94 ± 0.03** | 1.01 ± 0.04 |
| **2.5** | 1.00 ± 0.04 | 1.01 ± 0.08 | 1.02 ± 0.01 | 0.98 ± 0.05 | **0.92 ± 0.01** | 1.04 ± 0.04 |
| **5** | 1.01 ± 0.02 | 0.97 ± 0.02 | 1.03 ± 0.00 | 0.97 ± 0.00 | **0.93 ± 0.01** | 0.98 ± 0.05 |
| **7.5** | 1.04 ± 0.03 | 0.99 ± 0.09 | 1.00 ± 0.02 | 0.99 ± 0.02 | **0.92 ± 0.05** | 1.03 ± 0.06 |
| **9** | 1.02 ± 0.02 | 1.03 ± 0.01 | 1.03 ± 0.01 | 1.00 ± 0.01 | **0.86 ± 0.03** | 1.02 ± 0.00 |
| **21** | 0.98 ± 0.09 | 1.04 ± 0.02 | 1.00 ± 0.01 | 1.04 ± 0.02 | **0.81 ± 0.02** | 1.03 ± 0.01 |
| **27** | 0.98 ± 0.09 | 1.03 ± 0.01 | 1.04 ± 0.02 | 1.02 ± 0.01 | **0.82 ± 0.03** | 1.04 ± 0.02 |
|  | **TCPP-IS1** | | | **TCPP-IS2** | | |
| **Time (h)** | **Laccase** | **Laccase + ABTS** | **Laccase + medium** | **Laccase** | **Laccase + ABTS** | **Laccase + medium** |
| **0** | 1.00 ± 0.00 | 1.00 ± 0.00 | 1.00 ± 0.00 | 1.00 ± 0.00 | 1.00 ± 0.00 | 1.00 ± 0.00 |
| **1** | 1.00 ± 0.01 | 0.98 ± 0.01 | 1.00 ± 0.04 | 1.02 ± 0.03 | 0.97 ± 0.01 | 1.01 ± 0.04 |
| **2.5** | 1.00 ± 0.04 | 0.96 ± 0.01 | 1.04 ± 0.03 | 1.01 ± 0.06 | 0.96 ± 0.01 | 1.05 ± 0.03 |
| **5** | 0.99 ± 0.02 | 1.00 ± 0.05 | 1.05 ± 0.00 | 1.00 ± 0.02 | 1.01 ± 0.03 | 0.99 ± 0.00 |
| **7.5** | 0.96 ± 0.02 | 0.96 ± 0.02 | 1.03 ± 0.02 | 1.00 ± 0.02 | 1.00 ± 0.06 | 1.00 ± 0.04 |
| **9** | 1.02 ± 0.06 | 0.99 ± 0.01 | 1.03 ± 0.01 | 0.97 ± 0.01 | 0.96 ± 0.06 | 1.00 ± 0.05 |
| **21** | 0.96 ± 0.01 | 0.99 ± 0.02 | 1.01 ± 0.00 | 0.98 ± 0.03 | 0.99 ± 0.01 | 1.00 ± 0.06 |
| **27** | 0.97 ± 0.02 | 0.97 ± 0.02 | 1.03 ± 0.02 | 0.97 ± 0.01 | 0.98 ± 0.02 | 1.00 ± 0.07 |
|  | **TCPP-IS3** | | |  |  |  |
| **Time (h)** | **Laccase** | **Laccase + ABTS** | **Laccase + medium** |  |  |  |
| **0** | 1.00 ± 0.00 | 1.00 ± 0.00 | 1.00 ± 0.00 |  |  |  |
| **1** | 0.98 ± 0.00 | 0.98 ± 0.00 | 1.01 ± 0.06 |  |  |  |
| **2.5** | 0.97 ± 0.02 | 0.99 ± 0.01 | 1.04 ± 0.01 |  |  |  |
| **5** | 0.98 ± 0.05 | 0.99 ± 0.03 | 1.04 ± 0.00 |  |  |  |
| **7.5** | 0.97 ± 0.01 | 0.96 ± 0.02 | 1.00 ± 0.02 |  |  |  |
| **9** | 0.96 ± 0.04 | 1.00 ± 0.00 | 1.00 ± 0.02 |  |  |  |
| **21** | 0.98 ± 0.01 | 0.96 ± 0.03 | 1.01 ± 0.04 |  |  |  |
| **27** | 0.98 ± 0.01 | 1.02 ± 0.04 | 1.02 ± 0.03 |  |  |  |

**Figure S. 2:** Pseudo-first-order kinetics model for the degradation of the isomers of TCPP by T. versicolor pellets





**Figure S. 3:** Vibrio fischeri inhibition at 81.9% of the toxicants’ initial concentrations for each fungal candidate.
